# The Entrance Examinations in Dental Colleges

**Published:** 1897-10

**Authors:** 


### Editorial.

### THE ENTRANCE EXAMINATIONS IN DENTAL COLLEGES.

The severe criticisms made upon the action of the National Association of Dental Faculties, rescinding their decision made at Saratoga, 1896, and substituting a lower standard at Old Point Comfort the present year, have apparently a substantial basis. It is, however, very clear that this action is not understood; and while it would seem to be a retrograde movement, it is not so in any sense, but rather a conservative effort to reach even a higher standard, but not by the ill-considered methods adopted in 1896.

The history of the effort to advance the standard of preliminary training, as well as the curriculum of each of the dental colleges, has never been written, and probably never will be; but there has been a continued effort in the Association of Faculties for many years to increase the requirements, but at the same time arrange these so as to avoid undue friction with the elements with which it was forced to deal.

The primary object of this body is to advance the character and the work of all the dental schools; and in order to do this each must be a mutual help to the other, bearing, to a certain extent, the burdens of the weak, and discouraging the strong in any attempts to move faster than the former could safely follow.

In the present condition of our country it is recognized that dental colleges cannot all be of equal value, and it is equally apparent that the dependence upon some few centres of education, as was formerly the case, must be abandoned. The growth and necessities of the people of each State will eventually tend to a concentration of effort for the upbuilding of that State in all directions, and thus give its own citizens the advantage of home instruction. The time was when the medical schools of Philadelphia and New York were patronized by students from all parts of the United States. To a large extent this continues, but the difference between the past and the present is marked, so much so that while the number of matriculates has steadily increased, the number from outside States and Territories has regularly diminished, leading to the remark of a distinguished teacher of surgery,

"that the medical colleges of Philadelphia were becoming local institutions," meaning by this that the students were mainly derived from Pennsylvania.

The reasons for this change must be self-evident, and it is not necessary to enlarge upon them. States as well as individuals grow out of their swaddling-clothes, and will, sooner or later, demand an independent position; and as there is no monopoly of knowledge, or the power of imparting it, new colleges have sprung up over the land, covering all departments of human intelligence.

While this is true and a cause of congratulation, it is equally true that many of these newly formed colleges are not State institutions, and are relatively weak from lack of a proper endowment. They began largely as simple dental schools, and although chartered as colleges, with all the legal rights attached thereto, they are financially weak, and it becomes with them a struggle for existence.

It may be said by the critical that this means that they ought to die. This is not the view of the writer. There is not a dental school in existence to-day that has not gone through all the phases connected with a struggle upward, not excepting even those connected with universities; and, besides, outside of our large cities, these new colleges are needed, especially in the South and the far West, and every encouragement should be given these until they have finally laid their foundations strong enough to resist all destructive elements. It may be said, and with truth, that there is a limit to this multiplication of colleges, and that the Association of Faculties cannot forever be dragging the older institutions in the mire, in order to save these weaklings. It is true that there is a limit beyond which it will not be safe to go, and that limit is very nearly reached. When this time comes there will be no new colleges admitted as members, except they demand a standard equal to the best. In the mean time the dental colleges connected with universities, and those not so connected, but firmly established, can formulate their own standards, and thus set an example by demonstrating that a dental college can make its own terms and gain in respect and patronage by so doing. This has become a recognized fact in dental and medical departments and in higher colleges, so much so that it has become almost axiomatic that the higher the standard the larger the number of students.

When the National Association of Dental Faculties adjourned last year, 1896, it was with the comfortable feeling that the entrance examination had been raised to that of the freshman year in a high school. The final preparation of this was given to a committee to formulate and print in the Proceedings. This committee contained one gentleman of large experience in educational matters, but who was not a member of the Association of Dental Faculties. This training fitted him for general educational work, but naturally he could not comprehend the needs of a profession, and especially so recent a development as dentistry. The result of the work of the committee was a series of rules in which applicants for matriculation were required to reach a certain standard, and this was minutely detailed. When this report was published it was at once recognized that this was not what the National Association of Faculties had ordered; in fact, it was in excess of anything anticipated when authority was given the committee. The publication created general disturbance in college circles, for it was felt that the rules could not be carried out in the present existing conditions of dental education.

When the "Faculties" met at Old Point Comfort, the first thing that confronted the delegates was a determined opposition to these formulated rules, and this became so pronounced that nothing was left for the Association to do but to endeavor to retain the position of previous years of a standard equivalent to that required for entrance to a high school. It is very questionable whether this can be raised in the near future, but there is nothing to prevent individual dental colleges from advancing the entrance examinations to suit themselves.

The charge that the National Association of Dental Faculties took a backward step in 1897 is not true. It simply adopted, in plain terms, what it presumed had been adopted the previous year. The lesson learned was that it is never safe to leave any subject in the hands of a committee that requires the critical care of the entire body. Upon this rock many associations have gone to pieces, and that the Association of Faculties was safely landed in deep water without special injury is a cause of congratulation.

This conclusion has, however, no permanent features. The Association cannot afford to occupy this low position for any great length of time, and it becomes the duty of all dental colleges intrinsically weak to remember that this adjustment is only temporary, and that the stronger colleges cannot and will not much longer bear the burdens of the weak, but will insist that those who have sought fraternal association must continually struggle to meet the higher standard demanded by the increased intelligence and broader education in the scholarly circles of the world.

